# The year 2017 and the four-yearly evaluation of the Stricto Sensu Graduate Programs: investments and actions to continued progress

**DOI:** 10.1590/1518-8345.0000.2995

**Published:** 2017-12-21

**Authors:** Carmen Gracinda Silvan Scochi, Márcia de Assunção Ferreira, Francine Lima Gelbcke

**Affiliations:** 1 PhD, Coordenadora da Área de Enfermagem at Coordenação de Aperfeiçoamento de Pessoal de Nível Superior (CAPES), Brazil and Full Professor, Escola de Enfermagem de Ribeirão Preto, Universidade de São Paulo, PAHO/WHO Collaborating Centre for Nursing Research Development, Ribeirão Preto, SP, Brasil. E-mail: cscochi@eerp.usp.br; 2 PhD, Coordenadora Adjunta de Programas Acadêmicos da Área de Enfermagem at Coordenação de Aperfeiçoamento de Pessoal de Nível Superior (CAPES), Brazil and Full Professor, Escola de Enfermagem Anna Nery, Universidade Federal do Rio de Janeiro, Rio de Janeiro, RJ, Brazil. E-mail: marciassuncao@eean.ufrj.br; 3 PhD, Coordenadora Adjunta de Programas Profissionais da Área de Enfermagem at Coordenação de Aperfeiçoamento de Pessoal de Nível Superior (CAPES), Brazil and Associate Professor, Departamento de Enfermagem, Universidade Federal de Santa, Florianópolis, SC, Brazil. E-mail: fgelbcke@ccs.ufsc.br

**Figure:**
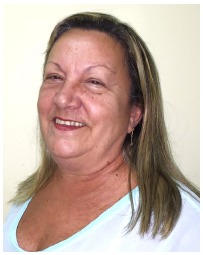

**Figure:**
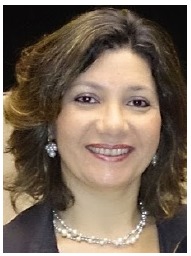

**Figure:**
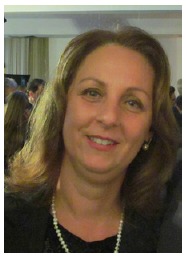

Stricto Sensu Graduate Nursing Programs in Brazil experienced innovations and achievements in 2017, both in terms of process plan and product of evaluation. Pioneerism with the interval of evaluation extended from three to four years, and the record and collection of data of the Programs through an *online* Platform open to the community, conferring agility and transparency to the process, are some remarks.

Furthermore, the criteria used for evaluation of Academic Programs were improved and better delineation, identity, productivity and evaluation process of Professional Master Programs were implemented, which were exhaustively discussed and constructed with peer collaboration in seminars and forums, in addition to the exercise of a partial evaluation of the Programs with wide debate of results in a Seminar entitled “Half Term Picture”. The incorporation of an evaluation process with a quality seal for the books produced in the Area (qualis Book) was also implemented in the quadrennium 2013-2016, joining the traditional process of qualification of periodicals in force.

Fifty-three academic programs and 21 professional master programs in operation in the five regions of the country were evaluated, showing an increase of nearly 30% in the present quadrennium, in relation to the 2010-2012 triennium, with a still asymmetrical distribution of courses, which are concentrated in the Southeast (42.9%), with advances in the Northeast (25.9%) and South (21.4%), and low numbers in the Midwest (8.0%) and North (1.8%)^1^.

In this group, professional master programs stand out for their remarkable expansion in the last six years, with a relative growth of 156%, representing 20.5% of the approved courses of the Nursing Area in December 2016. These courses target the training of professionals for health services and the consolidation of the Unified Health System^1^. 

In view of the strengthening of Nursing as Subject and Science, the expansion of the offer of PhD programs and interinstitutional academic cooperation initiatives became evident. With 38 doctoral programs, although none in the North of the country, the investments to reduce the asymmetries have been directed through academic solidarity, evidenced especially in the Federal and State Universities of Amazonas, Federal University of Acre and University of Rondônia, with the offer of special classes outside the Headquarter facilities, in the “Inter-institutional Doctoral program” (Dinter). 

The expansion of doctoral training has also been demonstrated beyond the borders of the country, reaching Latin America, with the assistance to implement Stricto Sensu Programs and reception of foreign professionals to conduct courses in the headquarters of the Programs. Solidary actions of international academic cooperation, such as the offer of a Dinter to PUC-Chile, and training of doctors to countries such as Mexico, Peru and Colombia, among others, strengthen the Nursing Area and the Brazilian Programs.

Since the implementation of the first course in 1972^2^, the Nursing Area has already trained 11,285 academic masters, 3,358 doctors and 786 professional masters; notably, 3,446 academic masters, 1,309 doctors and 631 professional masters qualified in the present quadriennium. These numbers tend to grow, in view of the annual presentation of projects for new course in response to the necessary preparation of doctors in Nursing in order to meet the goal of doubling the number of qualified researchers within 10 years, as established in the National Plan of Graduate Programs 2011-2020^3^.

The quantitative-qualitative growth of the Programs reverberated in the expressive scientific production evidenced in this quadrennium, which counted the publication of 16,321 articles, representing a relative growth of 77% in relation to the last triennium. The impact of this production can be seen in the international projection of Brazilian Nursing, in the jump from the eleventh position in the *ranking* of the Scopus/SCImago database in 2006 to the seventh in 2016 in terms of number of documents, overtaking the United States, United Kingdom, Australia, Canada, France and China. The challenge is to maintain stability in the *ranking* in citation of publications and raise the position in the H index, which has remained at 96, corresponding to the 22nd place^1^.

The international visibility of Researchers and Programs of the Nursing Area is expressed through various activities that take place in the framework of agreements, technical cooperation and other types of interinstitutional partnerships, as well as initiatives of incentive of research and events that increase mobility actions of researchers and students. These insertions have resulted in institutional academic ackowledgement, objectified in the *expertise* of professors who, as seen in the evaluation of the Programs, have worked on many and diverse work fronts in institutions, associations, societies, scientific journals and international events. 

Because these are programs whose goal is to qualify professionals, indicators to evaluate the insertion of new doctors in the labor market have also been applied in the evaluation of the Area and have evidenced the important work carried out in the field of *stricto sensu* postgraduate education, financed research, relevant positions in the management/headship of services and academia, among others. The data have shown the good placement or mobility of graduates with a rise in the labor market.

Internationalization has been the target of Nursing training, with expansion of international collaborative networks, exchanges of professors and students, and attraction of foreign professionals for qualification in Brazil, both in master’s and doctoral courses, reaffirming the excellence of the Programs of the Area. In the meantime, the scientific production of international circulation is the one that most strongly evidences the capillarity of diffusion and recognition of the Nursing Science produced in Brazil, so much that 40% of the weight of the evaluation of potential programs classified as 6 and 7 regarding international excellence is attributed to this aspect, followed by international participation (25%) and complemented by the analysis of indicators of faculty leadership (15%), nucleation (10%), and solidarity (10%)^1^.

When this scientific production is done in partnership with foreign researchers, it distinguishes and discriminates the international excellence of the Programs. In this quadriennium, among the 3,198 articles produced by the seven programs to which international excellence (6 and 7) was assigned, 10.7% were published with foreign authors.

Besides increased scientific production, the number of qualified journals in which the Nursing Programs have published increased by 30%, from 1,213 in the previous triennium to 1,579 in the present quadriennium, 39% of them with JCR/WoS and 53% with H/SCImago index^1^. Nonetheless, the challenge of publishing in international journals persists. 

Despite the increase of collaborative and multicentric research in partnership with foreign researchers, with consequent increase in joint scientific production, this aspect of the internationalization process still needs to improve, as well as the development of technological production in the Nursing Area, co-advising, double degrees, provision of classess in the English language and the raising of funds in foreign agencies/institutions. These represent challenges to be overcome in the coming years, requiring a strategic plan with actions to be implemented by the Programs.

In the process of evaluation of the present quadrennium, there were pioneerisms, as said, but portrayed achievements in itself, in the overcoming of adversities of the contemporary world, of shortage of funding, and of the impacts of retirement plans that imply the reformulation of the teaching staff. Nonetheless, the Area is aware of the challenges to be faced and remains firmly committed to advancing in training and evaluation processes that cause impacts with quality, applicable, and change-generating academic-scientific products.
